# Natural product ligands of FKBP12: Immunosuppressive antifungal agents FK506, rapamycin, and beyond

**DOI:** 10.1371/journal.ppat.1011056

**Published:** 2023-01-12

**Authors:** Angela Rivera, Joseph Heitman

**Affiliations:** 1 Department of Pharmacology and Cancer Biology, Duke University Medical Center, Durham, North Carolina, United States of America; 2 Department of Molecular Genetics and Microbiology, Duke University Medical Center, Durham, North Carolina, United States of America; 3 Department of Medicine, Duke University Medical Center, Durham, North Carolina, United States of America; University of Maryland, Baltimore, UNITED STATES

## Overview

Microorganisms produce natural products as a means of combating competing microorganisms and predators in the soil microenvironment. Modern medicine harnesses these naturally occurring compounds as bioactive agents for drug development. FK506-binding proteins (FKBPs) are enzymes that catalyze *cis*-trans peptidyl-prolyl isomerization, a key step during protein folding and function. FKBPs are conserved across eukaryotes and can bind natural products to form complexes that inhibit intracellular targets including calcineurin, TOR, and the centrosome-associated protein CEP250. The specificity with which these natural products bind the ubiquitous FKBPs to form protein-drug complexes with exquisite specificity for their targets paved the pathway to develop FK506 (tacrolimus) and rapamycin (sirolimus) and their analogs (pimecrolimus, everolimus, temsirolimus) as FDA approved drugs for transplant recipients, cancer chemotherapy, dermatology, and interventional cardiology. Additionally, because the organisms producing FKBP12 ligands are resident in soil, where natural products can be deployed for survival, this further illustrates why these ligands have potential for development as antimicrobial agents. The goal of this review is to highlight the known and unknown targets of natural product FKBP12 ligands to take stock of advances and further promote research in this area.

## Natural ligands with known targets

### FK506 (tacrolimus)

**Fig 1:**
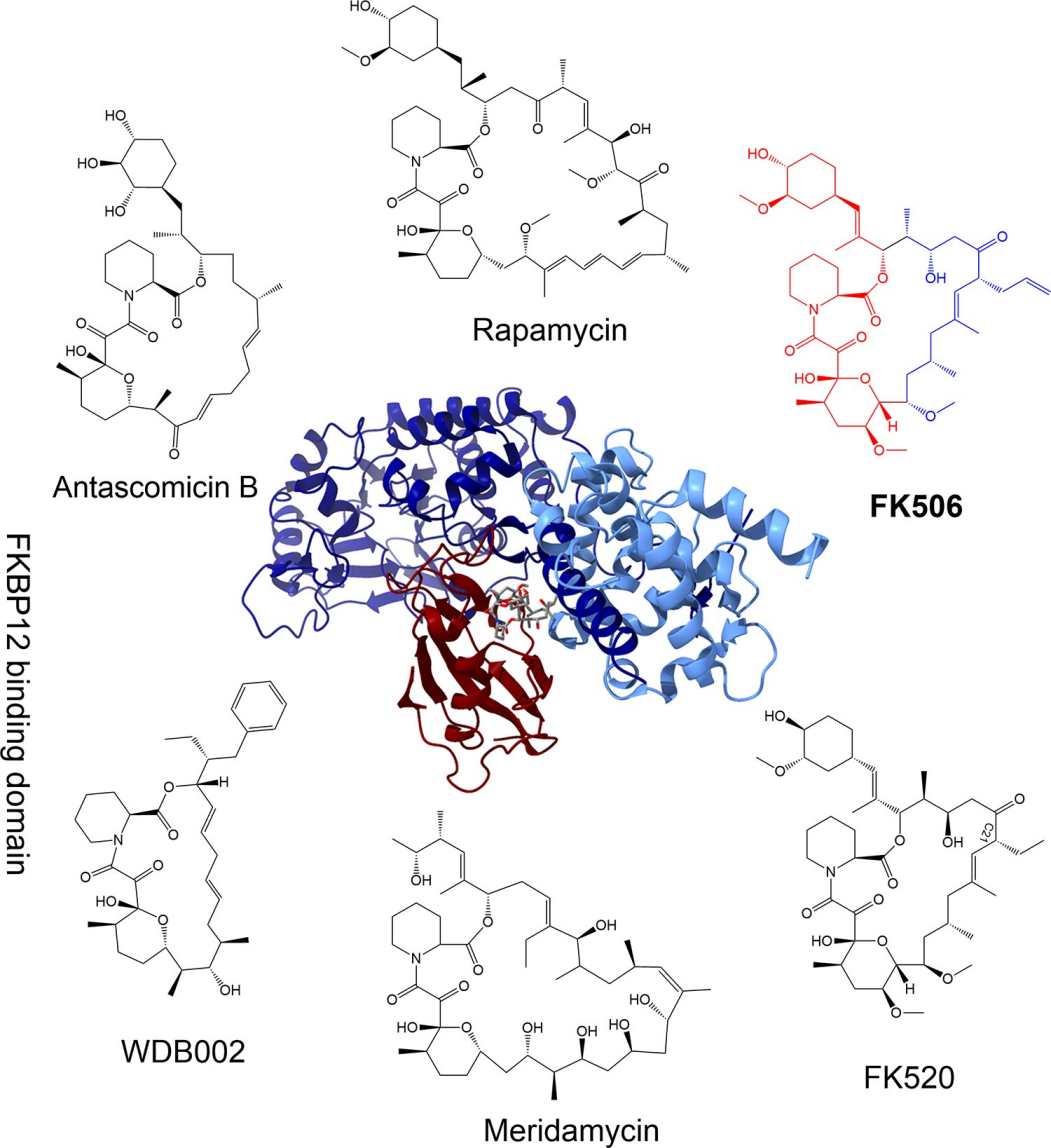
Chemical structures of FKBP12 ligands and crystal structure of FKBP12-FK506 inhibiting target calcineurin. Chemical structures include rapamycin, FK506, FK520, meridamycin, WDB002, and antascomicin B. FK506 chemical structure is shown with FKBP12 binding domain highlighted in red and the calcineurin binding domain highlighted in blue. In the center is the crystal structure of *Cryptococcus neoformans* calcineurin and FKBP12 bound to natural product FK506 (Calcineurin A subunit colored dark blue, calcineurin B subunit colored light blue, FKBP12 colored dark red, and FK506 colored by atom). Crystal structure adapted from “Harnessing calcineurin-FK506-FKBP12 crystal structures from invasive fungal pathogens to develop antifungal agents” (PDB 6TZ8) [3]. The original figure was published under CC BY 4.0 giving freedom to share and adapt figures from the source listed above.

FK506 is an FDA-approved immunosuppressive drug utilized to prevent and treat allograft rejection during organ and tissue transplantation. FK506 was originally isolated from *Streptomyces tsukubaensis* during a search for immunosuppressive compounds and was discovered to have potent immunosuppressive activity in both in vitro studies, such as in the mixed lymphocyte response assay, and also during in vivo murine studies [1]. The soil-resident bacterium *S*. *tsukubaensis* likely evolved to synthesize FK506 as a means to inhibit competitors and thereby enhance survival in the environmental niche. In fact, FK506 has been shown to inhibit growth of a variety of fungal species, some of which are also present in soil, thus supporting the hypothesis that *S*. *tsukubaensis* utilizes FK506 for competitive advantage [2,3,4]. The FKBP12-FK506 crystal structure from the human fungal pathogen *Cryptococcus neoformans* was recently elucidated in 2019 and is shown in Fig 1 [3]. The FKBP12-FK506-calcineurin crystal structure from humans has also been elucidated, and differences between the human and fungal proteins have been targeted to develop fungal-specific FK506 derivatives [3,5].

Based on a peptidyl-prolyl *cis*-*trans* isomerization assay that measures FKBP12 enzymatic activity, FK506 was found to bind FKBP12 and inhibit its function with an inhibition constant (K_i_) of approximately 1.7 nM [6]. Additionally, FKBP12 was independently identified as an FK506-binding partner through binding assays in extracts from a Jurkat T-cell line and the yeast *Saccharomyces cerevisiae* [7,8]. It was later found that the dissociation constant between FK506 and FKBP12 is 0.4 nM, demonstrating that FK506 has a high binding affinity in addition to potent inhibition of FKBP12 enzymatic activity [9].

The FKBP12-FK506 complex was found to inhibit calcineurin after the discovery of calcium-dependent FKBP12-FK506 binding in one of the first applications of glutathione S-transferase fusions for affinity purification via binding to immobilized glutathione [10]. Calcineurin binding by the FK506-FKBP12 complex was confirmed with anti-calcineurin antibodies as well as with in vitro and in vivo studies and was shown to have a direct impact on human T-cell signaling [10–12]. The FKBP12-FK506 complex was further shown to bind and inhibit calcineurin in fungi, demonstrating high conservation of the target of the FKBP12-FK506 complex [13,14].

### FK520 (ascomycin)

FK520 was originally isolated from *Streptomyces* KK317 and *Streptomyces hygroscopicus* subspecies *yakushimaensis* No. 7238, identified as an antifungal agent during an antimicrobial search from the producing strains, and later found to have immunosuppressive activity in studies searching for FK506 immunosuppressive analogs with lower toxicity [15,16]. FK520 is a structural analog of FK506 differing by a single allyl to ethyl group substitution at the C21 position and has a modest approximately 3-fold reduction in immunosuppressive and antifungal activity [4]. Similar to FK506, FK520 action requires FKBP12 as a binding partner, and the FKBP12-FK520 complex inhibits calcineurin [17].

FK520 also has antimalarial activity against the protozoan parasite *P*. *falciparum*, and *P*. *falciparum* is susceptible to FK506 and rapamycin [17,18]. Interestingly, the FKBP12 ortholog in *P*. *falciparum* acts as a protein chaperone in addition to exhibiting canonical peptidyl-prolyl isomerization activity [19]. Studies focusing on FK520 show that antimalarial activity is distinct from the FK520 immunosuppressive activity. This was demonstrated by modifications to the structure of FK520 that resulted in analogs with affinity for FKBP12 but not calcineurin. The resulting nonimmunosuppressive compounds were still potently inhibitory against *P*. *falciparum*, providing evidence the mode of action may involve FKBP12 but not calcineurin [17].

### Rapamycin (sirolimus)

Rapamycin was originally identified as a natural product of the soil-resident species *S*. *hygroscopicus* that exhibits potent antifungal activity against *Candida albicans*, *Microsporum gypsum*, and *Trichophyton granulosum* [20]. Rapamycin has also been found to inhibit growth of *S*. *cerevisiae*, *C*. *neoformans*, *Aspergillus fumigatus*, and *Mucor circinelloides*, some of which also reside in the soil in nature [21–23].

In complex with FKBP12, rapamycin inhibits its target protein TOR (Target of Rapamycin). FKBP12 loss of function mutations, including mutations in the FKBP12-active site/drug-binding pocket, result in resistance to rapamycin in *S*. *cerevisiae*, *C*. *albicans*, and *C*. *neoformans* [22,24]. Additionally, TOR was identified as the target of the FKBP12-rapamycin complex through genetic analysis of drug-resistant mutants in *S*. *cerevisiae* where spontaneous fungal mutants resistant to rapamycin were isolated. Mutations responsible for resistance were identified in the genes encoding Tor1, Tor2, and FKBP12 [5,8].

In humans, the FKBP12-rapamycin complex inhibits the mammalian TOR ortholog (mTOR), and treatment with rapamycin exerts an immunosuppressive effect [25,26]. Specifically, rapamycin inhibits T-cell proliferation in response to interleukin 2 (IL-2) and also impacts innate and adaptive immune responses [27]. Treatment with rapamycin is known to induce autophagy in *S*. *cerevisiae* and mammalian systems [28,29].

Due to their shared requirement of binding to FKBP12 for activity, rapamycin and FK506 can compete with each other as reciprocal antagonists [30]. The inhibition of T-cell receptor signaling by FK506 is competitively antagonized by excess rapamycin, and the inhibition of T-cell response to IL-2 by rapamycin is competitively antagonized by excess FK506. This antagonistic activity was further confirmed biochemically in affinity assays where rapamycin was found to displace FK506 from human FKBP12 and in studies in the yeast *S*. *cerevisiae* [8,30–32].

### WDB002

The most recently discovered natural FKBP12 ligand, WDB002, was identified in 2020 as a potential FKBP12-binding partner when the genomes of *Streptomyces malaysiensis* isolates were analyzed for gene clusters similar to those involved in the biosynthesis of rapamycin and FK506. During this search, a series of natural products was identified including WDB001, WDB002, and WDB011. These natural products have a conserved structural region, and crystal structures of the FKBP12-WDB002 complex demonstrate that WDB002 binds in the hydrophobic pocket of FKBP12. WDB002 makes hydrogen bonding contacts similar to those observed in the FKBP12 complexes with FK506 and rapamycin, and this results in high-affinity binding to FKBP12 with a K_D_ of approximately 4 nM. The target of the FKBP12-WDB002 complex was shown to be CEP250, a centrosome-associated protein. CEP250 was characterized as the target of FKBP12-WDB002 through an affinity-based protein assay, and the FKBP12-WDB002 complex binds CEP250 with an approximate binding affinity of K_D_ = 40 nM. In humans, CEP250 is a centrosome-associated protein, and treatment with WDB002 has been shown to impact chromosome organization and segregation in USO2 human cells. Although WDB002 has not yet been characterized to have antimicrobial activity, WDB002 is under clinical investigation as an antiviral due to CEP250 interaction with a SARS-CoV-2 protein [33].

## Natural ligands with unknown targets

### Antascomycin

Antascomycins A through E were identified from *Micromonospora n*. *sp*. *A92-306401* through an assay monitoring displacement of FK506 bound to human FKBP12. *Micromonospora n*. *sp*. *A92-306401* is a fermentative bacterial strain isolated from soil [34]. The antascomycins were purified through column chromatography, including silica gel and Sephadex, and shown to bind FKBP12 with a range of affinities similar to those of FK506 and rapamycin. However, in contrast to FK506, the antascomicins do not inhibit IL-2 production by T-lymphocytes. In contrast to rapamycin, the antascomycins do not inhibit T-cell proliferation in a mixed lymphocyte reaction and therefore are not thought to have immunosuppressive activity [34]. At this point, no other cellular activities have been characterized for antascomicins and no target of the FKBP12-antascomicin complexes has been identified.

### Meridamycin

Meridamycin was isolated from the soil bacterium *S*. *hygroscopicus* during a search for new immunomodulatory compounds. Meridamycin inhibits FK506 binding to FKBP12 in a competitive binding assay with an IC_50_ of 1 ng/mL. Meridamycin is nonimmunosuppressive and antagonizes both FK506 and rapamycin immunosuppressive activity in murine T cells [35]. Compared to FK506 and rapamycin, the FKBP12-binding domain of meridamycin shows conserved structural characteristics; however, no target has been identified for the FKBP12-meridamycin complex. The meridamycin biosynthetic gene cluster has been studied for genetically engineered modification, and an expression system for the synthetic pathway has been developed in *Escherichia coli* [36,37]. In 2016, four congeners were identified and named meridamycin A, B, C, and D [38]. When meridamycin and its congeners were tested for antimicrobial activity, only the original meridamycin showed activity against the soil dwelling-bacterium *Bacillus subtilis* [38].

## Discussion

Several natural products bind FKBP12 to form a complex capable of inhibiting their respective targets, including FK506, rapamycin, and WDB002. Organisms naturally producing these compounds likely utilize them to compete and enhance survival in their environmental niches. In fact, several of these compounds were isolated from the soil-dwelling bacterium *Streptomyces*. Fungal pathogens including *Aspergillus* and *Cryptococcus* species are also soil-dwelling microorganisms and are susceptible to several of these natural products. Water-resident *P*. *falciparum* is also susceptible to growth inhibition from some of these ligands, and these findings further support the hypothesis that producing organisms deploy these FKBP12 ligands for competitive advantage. Therefore, it is logical to consider that antimicrobial therapeutics might be developed from these natural product compounds [39]. However, the immunosuppressive activity of FK506 and rapamycin precludes their use for the treatment of fungal infections in immunocompromised patients, and current studies therefore seek to identify modified natural ligands that are potent antifungal agents with diminished or abolished immunosuppressive activity.

Two such ligands include meridamycin and antascomycin, but lack of commercial availability has limited research on these two ligands and precluded discovery of their possible targets. It will be of great scientific value to identify potential targets of the FKBP12-antascomycin and FKBP12-meridamycin complexes, such as through binding affinity screens as with recently identified WDB002. Complementary approaches to define their potential novel targets could include the following: (1) genetic selection of drug-resistant fungal mutants and whole genome sequence analysis; (2) analysis of overexpression libraries to identify genes conferring resistance in susceptible fungi; or (3) haploinsufficiency profiling/homozygous profiling (HIP-HOP) studies with *S*. *cerevisiae*. It is possible these ligands may bind additional FKBPs to form protein-drug complexes that have novel targets. Alternatively, their sole function may be to bind and occupy FKBP12 and protect against FK506 or rapamycin, either to protect the producing microbe or to protect microbes of the surrounding flora. However, the potential to identify novel antimicrobial compounds secreted by environmental microorganisms represents an exciting avenue for continued drug target and drug discovery.
